# *Mycobacterium mageritense*-associated refractory cutaneous infection and lymphadenitis in an immunocompetent adult: insights from genomic sequencing

**DOI:** 10.1186/s41182-026-00904-y

**Published:** 2026-01-16

**Authors:** Shinnosuke Fukushima, Jumpei Uchiyama, Yoshio Kawakami, Yoshiko Matsuura, Satoru Sugihara, Shin Morizane, Poowadon Muenraya, Hideharu Hagiya

**Affiliations:** 1 Department of Bacteriology, Dentistry and Pharmaceutical Sciences, Okayama University Graduate School of Medicine, Okayama, Japan; 2 Department of Infectious Diseases, Okayama University Hospital, 2-5-1 Shikata-Cho, Kitaku, Okayama, 700-8558 Japan; 3 Department of Dermatology, Okayama University Hospital, Okayama, Japan; 4 Konohana Dermatology Clinic, Okayama, Japan

**Keywords:** Genome sequence, Lymphadenitis, *Mycobacterium mageritense*, Skin and soft tissue infections, Rapidly growing mycobacteria

## Abstract

**Background:**

Nontuberculous mycobacteria are increasingly recognized as causes of chronic and refractory skin and soft tissue infections, even in individuals without immunodeficiency. Among them, *Mycobacterium mageritense* is a rare, rapidly growing species that can lead to persistent lesions requiring prolonged antimicrobial therapy. Reports of *M. mageritense* infections involving both the skin and regional lymph nodes are limited, and this case adds new clinical and genomic insights.

**Case presentation:**

A 48-year-old previously healthy man presented with a slowly enlarging cutaneous lesion on his lower leg and ipsilateral inguinal lymphadenitis. Empirical antibacterial therapy with β-lactams and macrolides was ineffective. Wound cultures subsequently grew *M. mageritense*, confirmed by whole-genome sequencing. Several antimicrobial regimens were attempted, and the final successful therapy consisted of oral levofloxacin and minocycline for 4 months, leading to complete clinical resolution. Genomic analysis identified resistance-related genes, including *erm(40)*, *aac(2′)-Ib*, *tet(V)*, and *RbpA*, although in vitro minimum inhibitory concentrations showed variable susceptibility. Phylogenetic comparison revealed that the isolate was closely related to previously reported *M. mageritense* strains from Japan.

**Conclusions:**

This case demonstrates that *M. mageritense* can cause cutaneous infection with secondary lymphadenitis in an immunocompetent host. Accurate species identification using molecular or genomic methods and selection of appropriate combination antibiotic therapy based on susceptibility testing are crucial for successful management of such infections.

## Introduction

*Mycobacterium mageritense* was first identified as a rapidly growing mycobacterium (RGM) in 1997, which is commonly found in water and soil [1–5]. The first clinical cases of *M. mageritense* infection*,* including surgical site infections, were reported in 2002, and since then, increasing numbers of cases, such as skin and soft tissue infections (SSTIs), lymphadenitis, and pulmonary diseases, have been reported [3, 6–9]. Respiratory infections have been reported in patients with immunosuppression, such as those receiving systemic steroid therapy, but SSTIs were diagnosed in immunocompetent individuals [6, 8–10]. *M. mageritense* strains generally show high minimum inhibitory concentration (MIC) for macrolides, which are often used in the initial treatment of nontuberculous mycobacteria (NTM) infections. This is explained by the fact that they possess the *erm(40)* gene (inducible erythromycin methylase 40 gene), which represents a characteristic mechanism for the development of macrolide resistance [11, 12]. Since quinolone monotherapy carries the risk of developing mutational resistance, combination therapy with minocycline (MINO) or sulfamethoxazole–trimethoprim (TMP–SMX) has been clinically reported [9, 13, 14]. Here, we describe a case of refractory cutaneous infection with *M. mageritense* in an immunocompetent adult patient, along with the results of whole-genome sequencing analysis for the present isolate.

## Case presentation

**Fig. 1:**
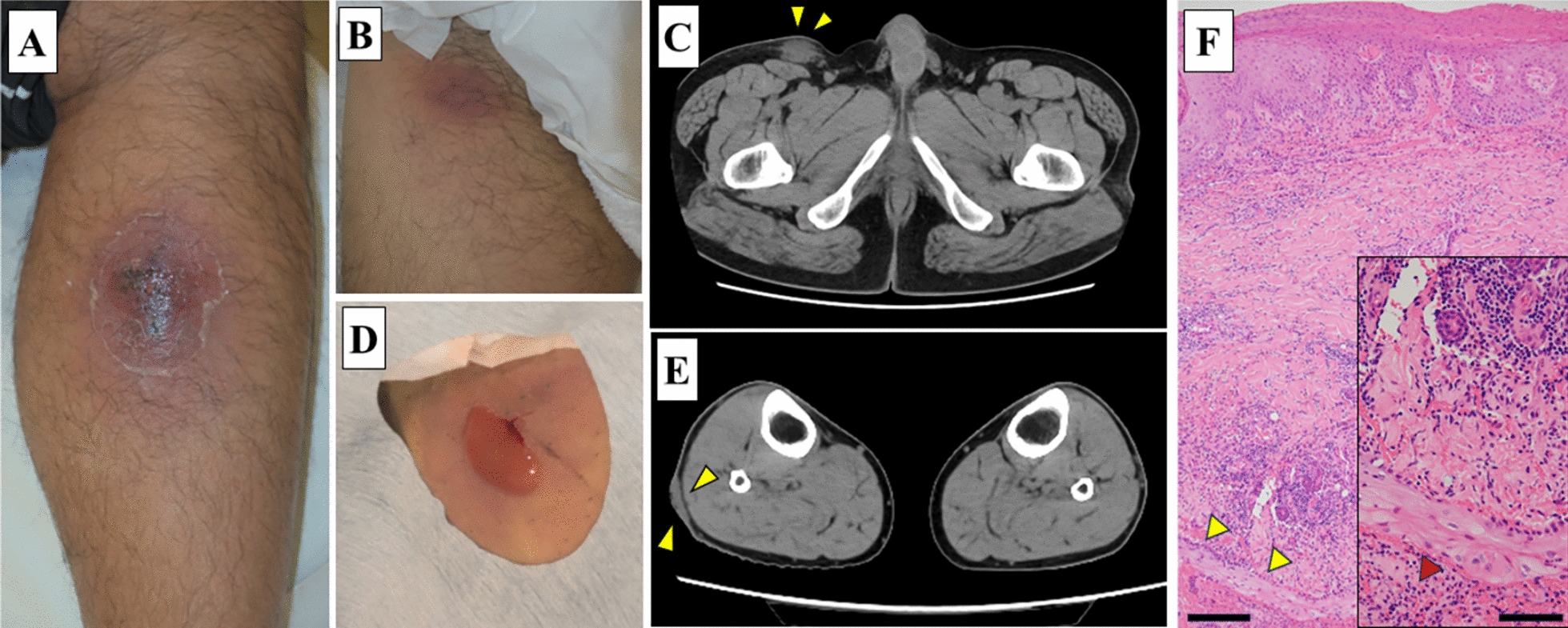
Image of findings. **A** 7 × 6 cm cutaneous abscess with a reddish surface and exudation. **B** Reddish and swelling lesion in the right inguinal region. **C** Computed tomography showing lymphadenitis in the right groin (the arrowheads). **D** Red and cloudy pus discharge through incision at the right inguinal region. **E** Computed tomography showing a cutaneous infection in the right posterior lower extremity (the arrowheads). **F** Hyperkeratosis and parakeratosis are observed in the epidermis layer. Inflammation caused mainly by lymphocytes is observed in the dermis layer, and fragments of stratified squamous epithelium without atypia are observed (the yellow arrowheads) (bar 200 μm). Neutrophil infiltration is observed under the squamous epithelium in inset (the red arrowhead) (bar 50 μm)

**Fig. 2:**
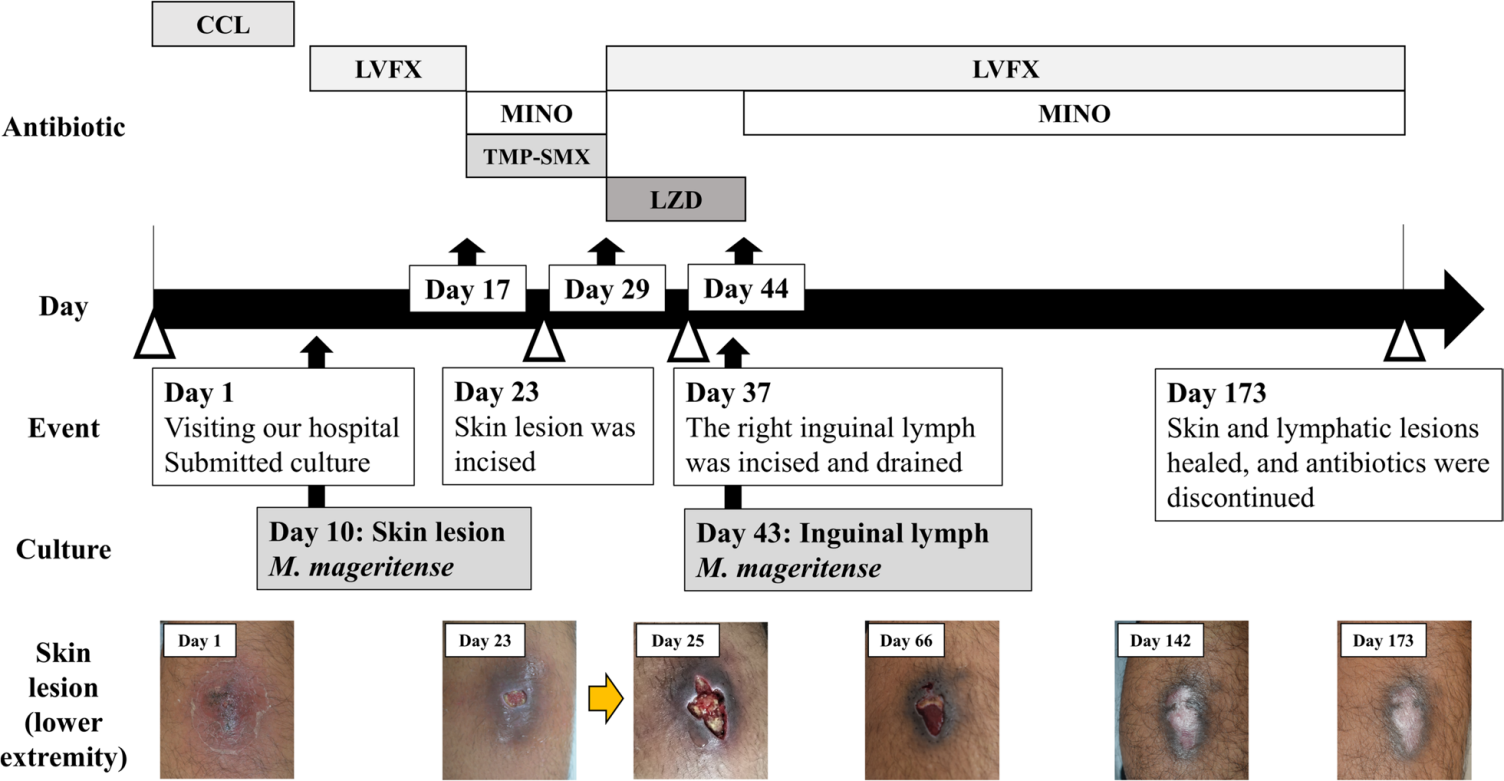
Clinical course of the case. *CCL* cefaclor, *LVFX* levofloxacin, *MINO* minocycline, *TMP–SMX* sulfamethoxazole–trimethoprim, *LZD* linezolid. The cutaneous lesion and inguinal lymph cultures identified *Mycobacterium mageritense*. The patient underwent oral antibiotic treatment for a total of 6 months, mainly with LVFX and MINO combination therapy for 4 months

A 48-year-old man without any underlying diseases presents with redness and swelling in his right lower extremity for a month without any preceding traumatic event. He was previously prescribed cefdinir for a cutaneous infection at a dermatology clinic, but the inflammation did not improve. He was then transferred to our hospital with a 7 × 6 cm reddish lesion with exudates on the posterior side of his right lower extremity (Fig. 1A). A wound culture from the refractory skin ulcer was collected, and the patient was initiated on cefaclor (CCL). Laboratory data showed a leukocyte count of 7,280/µL (reference range 3,300‒8,600/µL) with 68.2% of neutrophils (reference range 40‒70%), and a slight elevation of C-reactive protein level of 0.42 mg/dL (reference range < 0.15 mg/dL). The acid-fast bacillus culture using the mycobacteria growth indicator tube showed growth of acid-fast bacilli at day 10, which was identified as *M. mageritense* by matrix-assisted laser desorption ionization–time-of-flight mass spectrometry (MALDI Biotyper; Bruker Daltonics Inc., Billerica, MA, USA). No other bacterial pathogen was isolated. Therefore, CCL was switched to oral levofloxacin (LVFX). Additional examination revealed no diabetes mellitus (HbA1c 5.6%) or other obvious immunological impairment, including abnormalities in immunoglobulin levels, complement activities, and human immunodeficiency virus infection. Physical examination detected a satellite lesion in his right inguinal region (Fig. 1B), and computed tomography revealed a swollen lymph node measuring 30 × 17 mm (Fig. 1C), which was later drained by local incision (Fig. 1D). The cutaneous lesion in the right lower extremity did not involve the muscle (Fig. 1E). There were no other findings suggestive of systemic lymph node enlargement or disseminated lesions. The pathological finding of the cutaneous lesion showed neutrophil infiltration under squamous epithelium, and inflammation mainly caused by lymphocytes in the dermis layer, but no malignant findings (Fig. 1F). The patient was diagnosed with refractory cutaneous abscess caused by *M. mageritense* complicated with inguinal lymphadenitis. After *M. mageritense* was identified, the patient was treated with an oral combination of TMP–SMX (TMP 160 mg/SMX 800 mg, twice daily) and MINO (100 mg, twice daily). We refrained from administering fluoroquinolones, because he had a history of Achilles tendon rupture 2 years ago. Subsequently, antimicrobial susceptibility testing showed resistance to TMP–SMX (MIC, 80 μg/mL), and we changed to an oral combination of linezolid (LZD) (600 mg, twice daily) and LVFX (500 mg, once daily) on day 29 (Table 1). TMP–SMX and MINO were administered for a total of 13 days until this change. As little improvement was observed in the inguinal lymphadenitis following treatment, the involved lymph node was fully incised on day 37 and its drainage. The drainage fluid also showed the growth of *M. mageritense*. After that, the patient developed general malaise, which was considered to be associated with 16 days of LZD therapy, so it was switched to oral MINO (100 mg, twice daily) on day 44. An oral combination of LVFX 500 mg once a day and MINO 100 mg twice a day was continued for 4 months, and the wound finally improved (Fig. 2). Oral antibiotic treatment continued for a total of 6 months until the complete resolution of the inguinal lymph node and skin lesions.

**Table 1:** Antimicrobial susceptibility testing of *Mycobacterium mageritense*

Antibiotics	MIC (μg/mL)	Susceptibility
Amikacin	16	S
Tobramycin	> 16	R
Imipenem	≤ 2	S
Faropenem	4	N.d
Meropenem	4	S
Levofloxacin	≤ 1	N.d
Moxifloxacin	≤ 0.25	S
Sitafloxacin	≤ 0.25	N.d
Sulfamethoxazole/Trimethoprim	80	R
Doxycycline	2	I
Linezolid	2	S
Clofazimine	1	N.d
Azithromycin	> 64	N.d
Clarithromycin	> 64	R

*MIC* minimum inhibitory concentration, *N.d.* not determined. Susceptibility testing was performed using BrothMIC RGM (Kyokuto Pharmaceutical Industrial Co., Ltd., Tokyo, Japan) based on the Clinical and Laboratory Standards Institute M24 2nd edition

## Microbiological analysis

We performed whole-genome sequencing analysis to identify the bacterial species, detect antimicrobial resistance genes, and compare the phylogenetic relatedness between the present strain and those previously deposited in the database. DNA was extracted from bacteria cultured in a Middlebrook 7H9 broth (Mycobroth, Kyokuto Pharmaceutical Industrial Co., Ltd., Tokyo, Japan) at 30 °C for 24 h, using an ISOSPIN Fecal DNA Kit (Nippon Gene Co., Ltd., Tokyo, Japan). The Illumina NovaSeqX platform was used to sequence paired-end short-read sequencing libraries prepared using the NovaSeqX Series 10B Reagent Kit (Illumina, Inc., USA). The draft genome was obtained by trimming the sequence data using the software package fastp v0.23.2, followed by genome assembly using the SPAdes genome assembler. The genome was annotated using the prokka software v1.14.6. The antimicrobial resistance genes were predicted using two software such as the ABRicate software v1.0.1 and Resistance Gene Identifier software v6.0.5 on the Comprehensive Antibiotic Resistance Database v4.0.1 [15, 16]. Genome analysis was conducted using the Roary software v3.11.2, employing sequence data from the present case and five previously deposited data of *M. mageritense* strains [17].

**Fig. 3:**
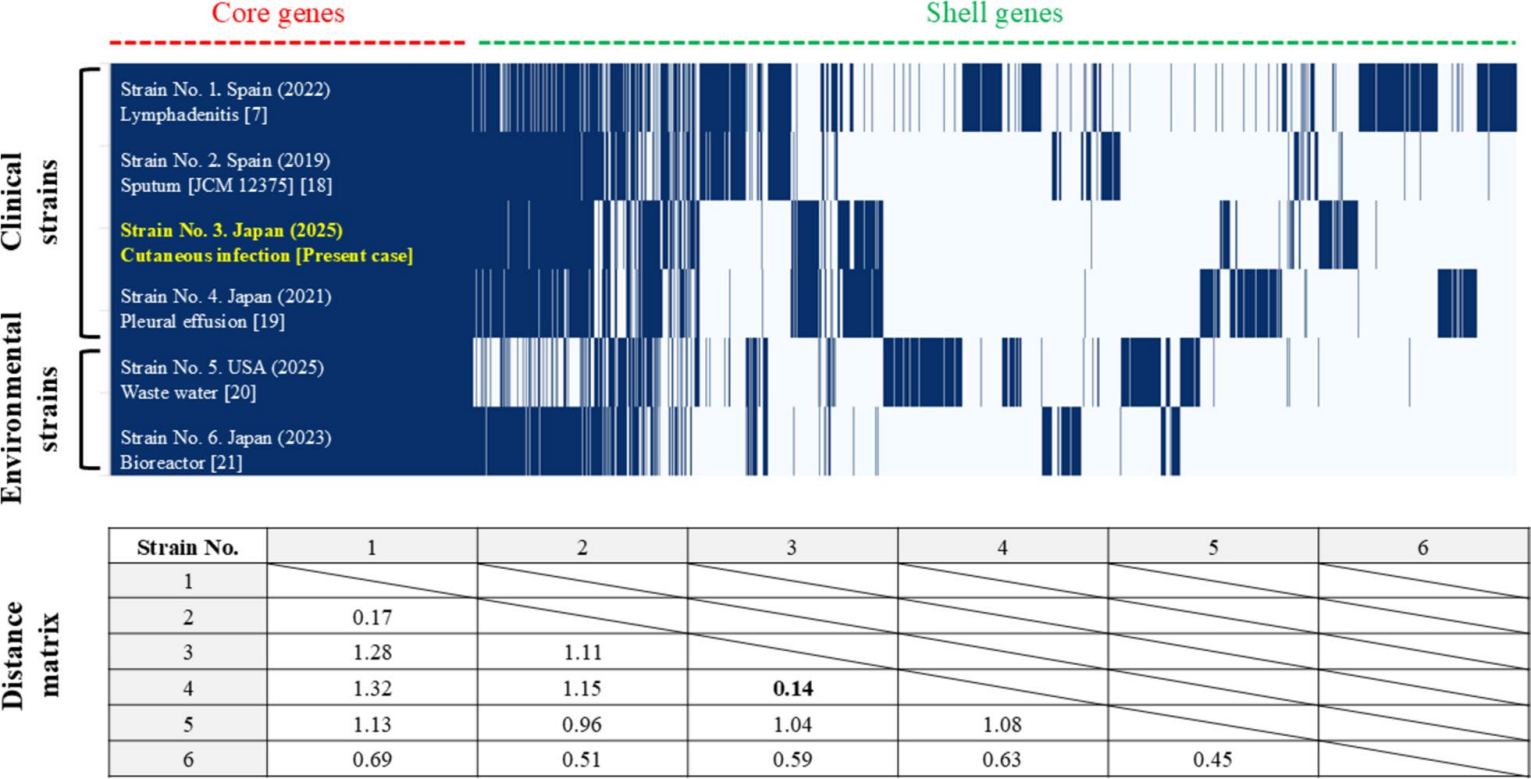
Genome sequencing analysis of *Mycobacterium mageritense* isolates. Phylogenetic relationship and gene presence/absence profiles of *M. mageritense* strains are generated using ROARY v3.11.2 based on a matrix of 11,798 cluster genes. The presence of gene matrix shows the distribution of annotated genes among the presented strain and five previously deposited *M. mageritense* strains obtained from GenBank. Each vertical blue bar represents the presence of a gene, while white spaces indicate its absence. Core genes are those present in all strains (99–100%), while shell genes are those found in many but not all strains (15–95%). From the distance matrix calculated based on the core genome alignment, the phylogenetic relatedness between strains can be determined. Lower values indicate closer evolutionary relationships, signifying greater genomic similarity between strains of interest

The draft genome was analyzed using the Type Strain Genome Server platform, showing the highest similarity to *M. mageritense* JCM12375 (d0 88.1% [84.7%‒90.9%], d4 91.3% [89.2%‒93.1%], d6 91.3% [88.7%‒93.3%], G + C content difference 0.09%). The following antimicrobial resistance genes were detected (% identity): *erm(40)* (associated with macrolide resistance; 98.8%), *aac(2')-Ib* (aminoglycoside resistance; 84.2%), *tet(V)* (tetracycline resistance; 81.2%), and *RbpA* (rifampicin resistance; 97.3%). Phylogenetic correlation among the five *M. mageritense* strains is depicted in Fig. 3 [7, 18–21]. Distance matrix between the present isolate and that detected in pleural effusion sample from a Japanese patient was the minimum at 0.14, suggesting the most closely related strains [19]. Those found in clinical samples in Spain also appeared similar to each other, with the distance matrix of 0.17 [7, 18]. The genome data were deposited in the GenBank, with an accession number of DRR705878.

## Discussion

*M. mageritense* is a non-pigmented RGM, accounting for 3.0‒7.1% of non-pigmented RGM isolates in a previous study [22]. This pathogen is commonly present in water and soil, occasionally causing SSTIs through contaminated water [4, 5, 11, 23]. Cutaneous NTM infections most commonly affect the lower extremities (50.9% of the cases), with a median age of disease onset of 51 years [24]. The risk factors include skin trauma and immunosuppression; notably, 56.8% of immunocompetent individuals with cutaneous NTM infection report a history of preceding trauma [24]. Cutaneous mycobacterial infections spread via the lymphatic system [25], but lymphadenitis caused by *M. mageritense* infection has been rarely documented. In the present case, *M. mageritense* was identified both in the cutaneous lesion in the lower extremity and the ipsilateral inguinal lymph node, suggesting dissemination through the lymphatic system. Notably, the present case had no history of trauma, underscoring the need to consider NTM infection and secondary lymphadenitis even in immunocompetent individuals without identifiable risk factors.

*M. mageritense* intrinsically harbors the *erm(40)* gene that confers resistance to macrolides, which are the key drugs for NTM infections [7–10, 21]. Therefore, quinolone-based combination therapy has been preferred, with prolonged treatment periods of 3‒6 months [7–10, 26]. In a pediatric patient with subcutaneous abscesses, tosufloxacin and LZD were administered for 6 months based on the results of susceptibility testing [9]. A tattoo-associated case was treated with moxifloxacin and MINO for 3 months [7, 21]. Notably, several cases have been successfully treated with clarithromycin (CAM)-based combination regimen, despite the presence of the *erm(40)* gene [7, 26]. Earlier case reports suggested that *M. mageritense* isolates would not necessarily show resistance to macrolides; *e.g.,* one clinical isolate showed a lower MIC value to CAM (0.25 μg/mL) [26], while another exhibited a very high MIC (> 16 μg/mL) [22]. Susceptibility to macrolides may rely not solely on *erm(40)* but also on other mechanisms.

Three other drug resistance genes were identified in our isolate. The *aac(2')-Ib* gene confers resistance to aminoglycosides, such as gentamicin and tobramycin, which has been reportedly identified in other RGM isolates, such as *Mycobacterium smegmatis* [27]. The *tet(V)* gene reportedly codes for a drug antiporter which uses the proton motive force for the active efflux of tetracycline in *M. smegmatis* and *Mycobacterium fortuitum* [28]. The *RbpA* gene, encoding RNA polymerase (RNAP) binding protein A (RbpA) that contributes to the formation of stable RNAP–promoter open complexes, is related to rifampicin resistance [29, 30]. However, the *RbpA* and *tet(V)* genes are appropriately 80% identical and may be considered variants within the same family but may be functionally distinct. In our case, this case was successfully treated with a combination treatment regimen that included MINO regardless of the *tet(V)* gene.

In summary, we described a clinical case of *M. mageritense* infection that caused a refractory cutaneous infection and lymphadenitis in an immunocompetent adult, in which various antimicrobial resistance genes were identified. The clinical course of the patient was favorable with the combination treatment of LVFX and MINO. This case underscores the importance of accurate identification of *Mycobacterium* species, considering that *M. mageritense* is generally resistant to anti-tuberculous drugs and macrolide antibiotics commonly used to treat NTM infections. However, phenotypic resistance may vary from one isolate to another, and treatment strategy must be tailored to each individual case based on antimicrobial susceptibility testing.

## Data Availability

The data sets used during the current study are available from the corresponding author upon reasonable request. The data sets used during the current study are available from the corresponding author on reasonable request. The raw sequencing data were deposited in DDBJ Read Archive (accession no. DRR705878).
